# Molecular Structure of Amyloid Fibrils Controls the Relationship between Fibrillar Size and Toxicity

**DOI:** 10.1371/journal.pone.0020244

**Published:** 2011-05-20

**Authors:** Young Jin Lee, Regina Savtchenko, Valeriy G. Ostapchenko, Natallia Makarava, Ilia V. Baskakov

**Affiliations:** Department of Anatomy and Neurobiology and Center for Biomedical Engineering and Technology, University of Maryland School of Medicine, Baltimore, Maryland, United States of America; Creighton University, United States of America

## Abstract

**Background:**

According to the prevailing view, soluble oligomers or small fibrillar fragments are considered to be the most toxic species in prion diseases. To test this hypothesis, two conformationally different amyloid states were produced from the same highly pure recombinant full-length prion protein (rPrP). The cytotoxic potential of intact fibrils and fibrillar fragments generated by sonication from these two states was tested using cultured cells.

**Methodology/Principal Findings:**

For one amyloid state, fibril fragmentation was found to enhance its cytotoxic potential, whereas for another amyloid state formed within the same amino acid sequence, the fragmented fibrils were found to be substantially less toxic than the intact fibrils. Consistent with the previous studies, the toxic effects were more pronounced for cell cultures expressing normal isoform of the prion protein (PrP^C^) at high levels confirming that cytotoxicity was in part PrP^C^-dependent. Silencing of PrP^C^ expression by small hairpin RNAs designed to silence expression of human PrP^C^ (shRNA-PrP^C^) deminished the deleterious effects of the two amyloid states to a different extent, suggesting that the role of PrP^C^-mediated and PrP^C^-independent mechanisms depends on the structure of the aggregates.

**Conclusions/Significance:**

This work provides a direct illustration that the relationship between an amyloid's physical dimension and its toxic potential is not unidirectional but is controlled by the molecular structure of prion protein (PrP) molecules within aggregated states. Depending on the structure, a decrease in size of amyloid fibrils can either enhance or abolish their cytotoxic effect. Regardless of the molecular structure or size of PrP aggregates, silencing of PrP^C^ expression can be exploited to reduce their deleterious effects.

## Introduction

Etiology and progression of several neurodegenerative diseases including Alzheimer's, Parkinson's, Huntington's and prion diseases are linked to the accumulation of protein aggregates in the form of large amyloid fibrils/plaques, or small oligomers or fibrillar fragments [1]–[4]. According to the prevailing opinion, oligomers or small fibrillar fragments are the most toxic species and are responsible for the impairment of cellular functions, whereas mature fibrils or plaques are considered to be protective [3]–[7]. Small soluble oligomers could be produced as prefibrillar intermediates on the pathway to mature amyloid fibrils [8]–[11], as a result of fragmentation of mature fibrils or large aggregates [12], or as off-pathway products formed through alternative aggregation mechanisms [13]–[15]. Small oligomeric PrP particles produced by sonication from large pathogenic aggregates of the prion protein (PrP^Sc^) were found to exhibit the highest specific prion infectivity [16]. Aggregation of mature fibrils into deposits and plaques is considered to be a protective mechanism that evolved in nature to avoid the high intrinsic toxicity of soluble oligomers or small fibrillar fragments [3], [5], [17]. Defining the relationship between size, molecular architecture and toxicity of protein aggregates is essential for developing effective strategies for therapeutic intervention against neurodegenerative diseases.

The current studies were designed to test the hypothesis about the relationship between prion protein fibril dimension and their cytotoxic potential and specifically, to address the question of whether fragmentation of fibrils into smaller fragments or oligomers always enhances toxic potential. To address this question, two conformationally different fibrillar amyloid states referred to as R- and S-fibrils were produced from highly-pure, full-length Syrian hamster rPrP. The cytotoxic potential of intact fibrils and small fibrillar fragments generated by sonication was tested using cultured cells. For one amyloid state, fibril fragmentation was found to enhance its cytotoxic potential, whereas for another amyloid state formed within the same amino acid sequence, the fragmented fibrils were found to be less toxic than the intact fibrils. These studies show that molecular structure of the amyloid state controls the relationship between fibrillar size and toxicity.

## Results

**Figure 1:**
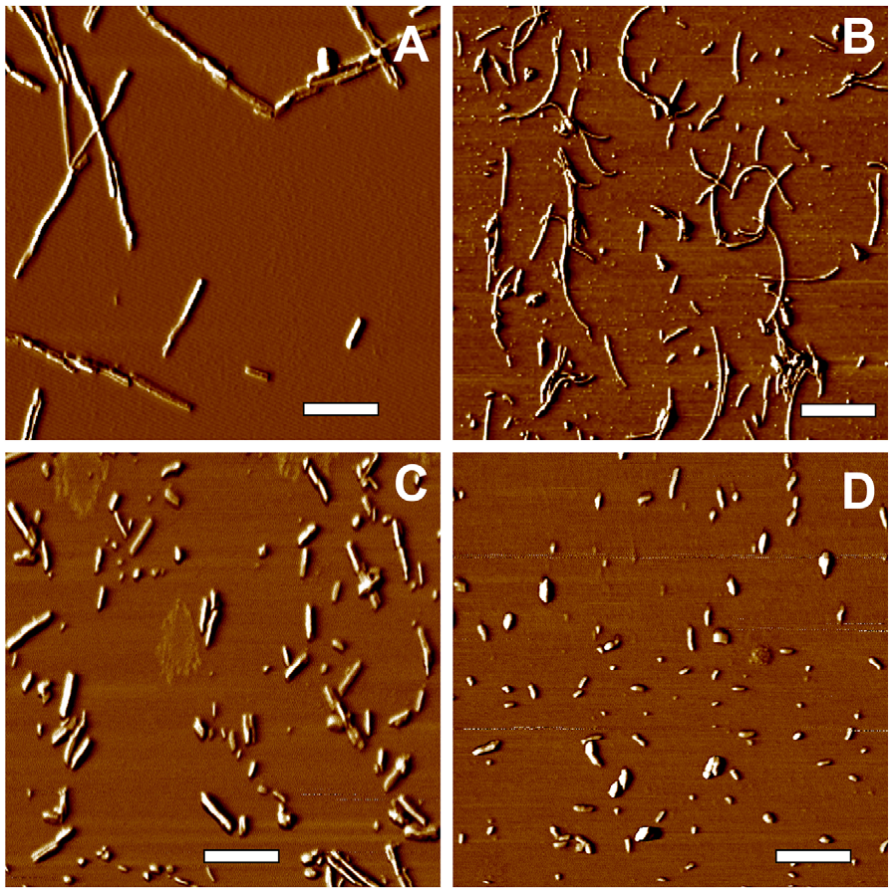
Atomic Force Microscopy imaging of R- and S-fibrils. Phase AFM images of intact R- and S-fibrils (A and B, respectively), or R- and S-fibrils after fragmentation by ultrasound treatment (C and D respectively). Scale bars = 0.5 µm.

The R- and S-fibrils were formed from full-length rPrP encompassing residues 23–231 under identical solvent conditions but different agitation modes as previously described [18]. To examine the relationship between physical size and cytotoxicity, R- and S-fibrils were fragmented using a well-controlled sonication procedure (Fig. 1) [12], and toxicities of intact and fragmented fibrils were tested using cultured cells. Importantly, after sonication, R- and S-amyloid states preserved their individual S- or R-specific conformations despite smaller particle size [19].

**Figure 2:**
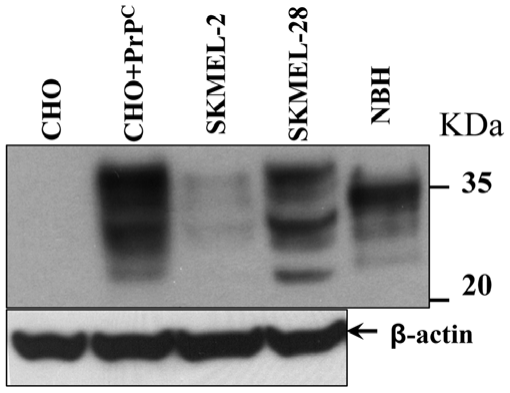
Analysis of PrP^C^ expression in CHO and SKMEL cell lines. CHO cells before transfection (CHO) or after transfection with pcDNA5/FRT/PrP plasmid (CHO+PrP^C^), and SKMEL-2 or SKMEL-28 cells (10^7^ cells for each cell line) were lysed and analyzed by Western blotting using mouse anti-PrP 3F4 or mouse anti-β-actin antibody. NBH – 10% normal hamster brain homogenate. β-actin was used as a loading control.

In our previous study, the cells of non-neuronal origin were found to exhibit the same ranking order in their susceptibility with respect to the toxic effect of different rPrP isoforms as cells of neuronal origin [20]. Because the toxic effects of extracellular PrP aggregates is known to be mediated by a surface-expressed PrP^C^ [20]–[24], in choosing the cell lines for the current study we were guided by the range of PrP^C^ expression but not by the cell type. We chose two SKMEL cell lines, SKMEL-2 and SKMEL-28 that express PrP^C^ at very low or high levels, respectively (Fig. 2). For the same reasons, Chinese Hamster Ovary (CHO) cells were used in parallel with CHO cells transfected with pcDNA5/FRT/PrP plasmid containing the gene for expression of wild type full-length Syrian hamster PrP^C^ (Fig. 2). Two assays were used for evaluating the effect of intact or fragmented R- or S-fibrils on cultured cells. The XTT assay assesses cell metabolism by measuring the activity of mitochondrial dehydrogenases, whereas trypan blue selectively stains dead cells.

**Figure 3:**
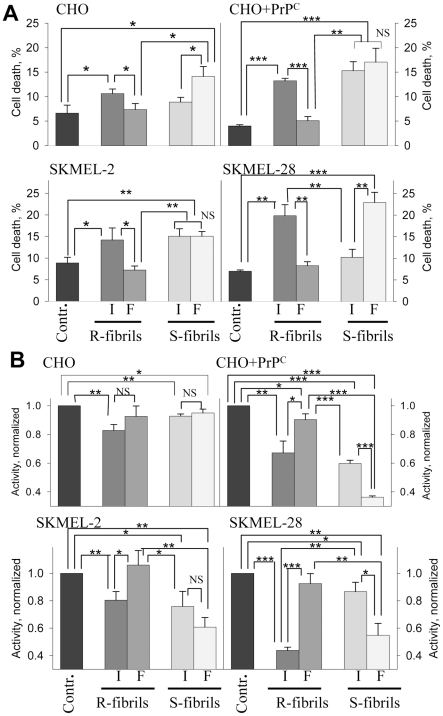
Analysis of cytotoxic potential of intact or fragmented R- and S-fibrils. Percentage of cell death (A) or activity of mitochondrial dehydrogenases (B) in CHO cells, CHO cells transfected with pcDNA5/FRT/PrP plasmid (CHO+PrP^C^), SKMEL-2, and SKMEL-28 cells as measured by Trypan Blue (A) or XTT (B) assays, respectively. Cells were seeded at 10^6^cells/cm^2^ density, cultured for one day prior to administration of rPrP fibrils (1 µM), and then for 24 hours after administration of intact (I) or fragmented (F) R- or S-fibrils. Contr – untreated controls. Each data set represents a mean value ± SD of three independent experiments for both assays. Approximately 500 cells were counted for each data point in each experiment for the Trypan Blue assay. In the XTT assay, XTT activities of untreated controls were set at 100% in each independent experiment. Statistical analyses were performed using Student's t-test. *P<0.05; **P<0.005; ***P<0.0005; NS, non-significant.

Comparative analysis of cell viability within each cell line and between lines revealed the following relationships (Fig. 3). First, the cell lines that expressed low levels of PrP^C^ (SKMEL-2 and non-transfected CHO cells) showed very modest cytotoxic responses. The toxic effects tend to be higher in the lines with high levels of PrP^C^ expression (SKMEL-28 and transfected CHO cells) (Fig. 3A,B). Second, contrary to the prevailing view, fragmented R-fibrils were found to be substantially less toxic than the intact R-fibrils in all cultured cells (Fig. 3A,B). In fact, as judged from both assays, fragmented R-fibrils had minimal effect, if any, as compared to the non-treated controls. Again, when treated with intact R-fibrils, the cellular response was weaker in lines with low levels of PrP^C^ expression. Third, the cytotoxic potential of S-fibrils followed the opposite trend (Fig. 3A,B): the toxicity of fragmented S-fibrils were similar or more pronounced as compared to intact S-fibrils. These findings revealed that small fibrillar fragments were more toxic than the intact fibrils for one amyloid structure, whereas for the alternative amyloid structure the small fragments were considerably less toxic than the intact fibrils. Interestingly, while fragmented R-fibrils were much smaller than the intact S-fibrils (Fig. 1), the former were generally less toxic than the latter (Fig. 3). This observation further supports the notion that size difference alone can not explain the toxic potential of amyloid states.

The differences in amplitude of response between the two assays were probably due to the fact that the XTT assay probes changes in the cell metabolism that could be considered as an intermediate step in a cell's response, whereas trypan blue assesses the percentage of cells that were irreversibly damaged and died. Regardless of the level of PrP^C^ expression, all cell lines showed similar rank orders with respect to their susceptibilities to intact or sonicated R- or S-fibrils within each individual cell line (Fig. 3A,B).

**Figure 4:**
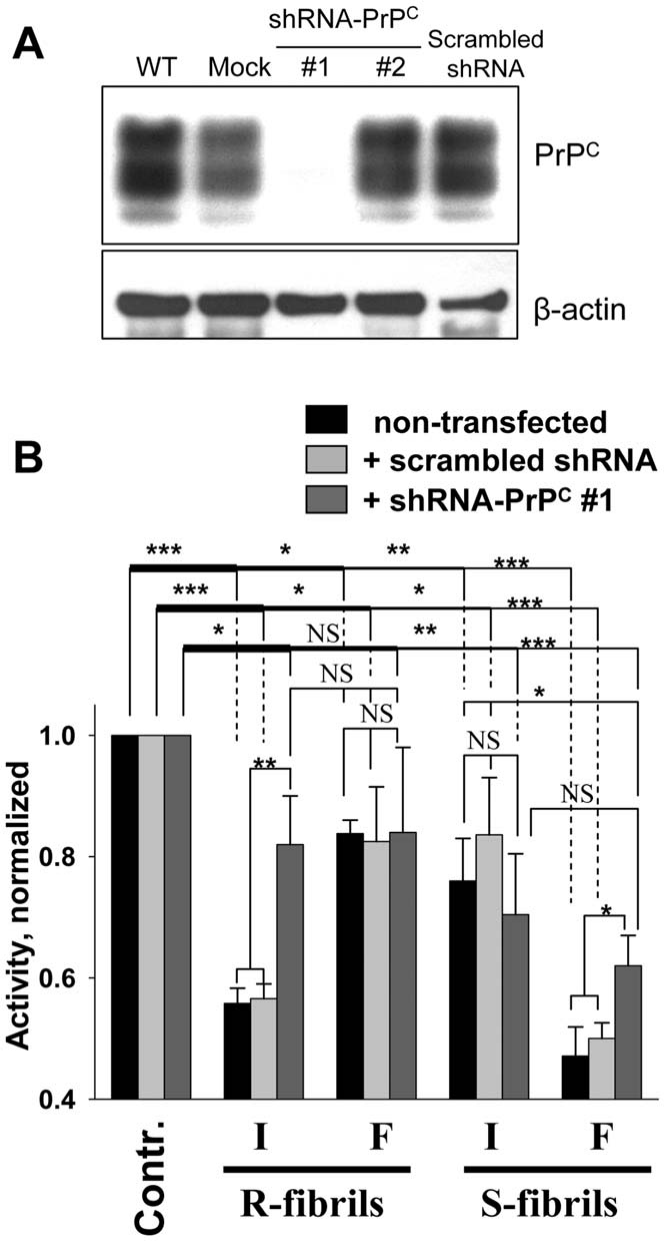
Effect of PrP^C^ silencing by shRNA-PrP^C^ on toxicity of the R- and S-fibrils. (**A**) Expression of PrP^C^ in SKMEL-28 cells (WT) and SKMEL-28 cells transfected with lentiviruses encoding shRNA-PrP^C^#1, shRNA-PrP^C^#2, or scrambled shRNA, or with empty lentiviral vector (Mock). β-actin was used as a loading control. (**B**) XTT assay of SKMEL-28 cells transduced with lentiviruses encoding shRNA-PrP^C^#1 or scrambled shRNA and treated with intact (I) or fragmented (F) R- or S-fibrils (1 µM). Each data set represents a mean value ± SD of three independent experiments. In the XTT assay, XTT activities of untreated controls were set at 100% in each independent experiment. Activities for each set of SKMEL-28 cells (non-transfected, transfected with scrambled shRNA or shRNA-PrPC#1 lentiviruses) were normalized relative to the corresponding controls. Statistical analysis was performed using Student's t-test. *P<0.05; **P<0.01; ***P<0.0005; NS, non-significant.

The differences in cellular response between CHO and CHO+PrPC or SKMEL-2 and SKMEL-28 were relatively minor, which could be due to the fact that both SKMEL-2 and CHO express low levels of PrP^C^ (PrP^C^ in CHO can be detected using polyclonal serum R073 [25]), To test the extent to which the toxic effects of intact R- or fragmented S-fibrils were mediated by PrP^C^, SKMEL-28 cells were transduced with lentiviruses carrying shRNAs that were designed to silence *Prnp*, the gene encoding PrP^C^ expression, via RNA interference. Two lentiviruses were constructed using shRNA-PrP^C^ vectors corresponding to two different segments within the 3′ UTR region of the human *Prnp* gene (will be referred to as shRNA-PrP^C^#1 and shRNA-PrP^C^#2). As judged from Western blotting, expression of PrP^C^ was silenced in cells treated with shRNA-PrP^C^#1 lentivirus, but not in cells transduced with shRNA-PrP^C^#2 or with scrambled shRNA lentiviruses (Fig. 4A). These differences could be attributed to the differences in sequences of shRNA-PrP^C^#1 and shRNA-PrP^C^#2 or differences in sites of chromosomal integration of two lentiviruses. To assess the role of PrP^C^, SKMEL-28 cells were transduced by shRNA-PrP^C^#1 or scrambled shRNA lentiviruses and then treated with intact or fragmented R- or S-fibrils (Fig. 4B). Consistent with previous data, non-transduced cells or cells transduced by scrambled shRNA lentivirus showed substantial reduction in mitochondrial activity after treatment with intact R-fibrils or fragmented S-fibrils (Fig. 4B). Cells treated with fragmented R- or intact S-fibrils exhibited very modest reduction in dehydrogenase activity in comparison to non-treated controls (Fig. 4B). Transduction of cells with shRNA-PrP^C^#1 lentivirus substantially reduced the deleterious effect of intact R-fibrils and partially diminished the effect of fragmented S-fibrils (Fig. 4B). However, silencing of PrP^C^ expression by shRNA-PrP^C^#1 had no measurable effects on cells treated with fragmented R- or intact S-fibrils, which both exhibited minor cytotoxic effects (Fig. 4B). These data supported the previous results obtained with cell lines that express PrP^C^ at different levels and revealed that the toxic effects of both R- and S-structures were mediated at least in part by PrP^C^. Considering that the mitochondrial activity in cells treated with shRNA-PrP^C^#1 lentivirus was not restored fully, there might be an alternative, PrP^C^-independent mechanism that mediates toxic signals of extracellular PrP aggregates.

## Discussion

Establishing the relationship between the physical state of a protein and toxicity is essential for developing effective therapeutic strategies against neurodegenerative diseases. In the prevailing opinion, soluble oligomers or small fibrillar fragments are considered to be the most toxic species, whereas formation of large amyloid fibrils and plaques are thought to be a protective process by which cells sequester more dangerous oligomers [3]–[7]. The present finding provides new opportunities for reexamining this view. This work warns that without specifying the molecular structures of the protein aggregates, claims regarding the relationships between size and toxicity of amyloid states could be misleading.

The current study revealed that for the S-structures produced from full-length recombinant prion protein, small fibrillar fragments were more toxic than the intact fibrils, whereas for the R-structures produced from the same protein, the small fragments were considerably less toxic than the intact fibrils. Remarkably, fragmentation of R-fibrils almost completely abolished their cytotoxic potential. Considering that both R- and S-fibrils are produced within the same amino acid sequence using highly pure rPrP [18], [19], the differences in cell response to intact versus fragmented fibrils have to be attributed to the distinct molecular structures of the two amyloid states. The R- and S- structures were analyzed previously using a broad range of biophysical techniques including X-ray diffraction, CD, hydrogen-deuterium exchange Raman spectroscopy, FTIR spectroscopy, hydrogen-deuterium exchange monitored by FTIR, proteinase K (PK)-digestion assay, binding of a conformation-sensitive fluorescence dye, immunoconformational assay, atomic force microscopy and electron microscopy [18], [19]. The R- and S-fibrils were found to have fundamentally different secondary, tertiary and quaternary structures [19]. While both amyloid states displayed a 4.8 Å meridional X-ray diffraction typical for amyloid cross-β spines, they showed markedly different equatorial profiles suggesting fundamentally different architectures of the cross β-spine [19]. Using solid state NMR, the cross-β core of R-fibrils was found to consist of in-register, parallel β-sheet structure [26]. No molecular details are currently available from NMR methods about structure of S-fibrils. Nevertheless, together with previous studies this work demonstrates that the relationship between fibril size and their cytotoxic potential is not unidirectional and is controlled by the molecular structures of the amyloid states.

The current work demonstrated the remarkable ability of cells to recognize and respond differently to conformationally distinct amyloid states even if they are formed within the same amino acid sequence. As evident from previous studies, not only were the cross β-spine structures markedly different in R- and S-fibrils, but also their surface epitope presentation and PK-resistant regions [18], [19]. For instance, the epitope to R1 antibodies (resides 225–231) was found to be solvent exposed in S-fibrils, but buried in the fibrillar interior in R-structures [18]. The N-terminal region 23–∼50 was found to be PK-resistant in S-structures, but PK-sensitive in R-fibrils [19]. As judged from the epitope presentation and PK-resistant profile, R-fibrils resembled the structure of PrP^Sc^ more closely than the S-fibrils. Moreover, unlike S-fibrils, R-fibrils were found to be capable of inducing a transmissible form of prion diseases in wild type animals [27]. Unexpectedly, fragmentation of R-amyloids into fibrils of shorter length was found to abolish their cytotoxic potential, an observation that contradicts the currently dominating view. We do not know whether the cellular response is controlled by R- or S-specific differences in their cross β-spines or by differences in the presentation of epitopes on lateral fibrillar surfaces.

In animals and human, prions target and replicate in cells of neuronal and non-neuronal origin in a variety of tissues [28]–[32]. As in the case for PrP^Sc^-induced toxicity [21]–[24], the toxic potential of the R- and S-amyloid structures was found to depend in part on the level of PrP^C^ expression. A growing number of studies illustrate that on cell surface PrP^C^ interacts with and mediates neurotoxic signaling of various β-sheet rich oligomers or fibrils formed by non-PrP proteins or peptides [33]–[35]. These findings suggest that PrP^C^ might be involved in mediating toxic signals in a number of neurodegenerative diseases. The results of the current studies support the idea that silencing of PrP^C^ expression offers a valuable therapeutic strategy as it limits the toxic effects of large fibrils or small fibrillar fragments. PrP^C^ dependence of the cellular response does not exclude the possibility that different signaling cascades are triggered by structurally different fibrils or particles, and that fibril-triggered toxicity might involve multiple mechanisms [36]. Sporadic Creutzfeldt-Jakob Disease is known to display substantial heterogeneity in neuropathological features including variations in lesion profile and PrP^Sc^ deposition [37], [38]. It would be difficult to explain the substantial phenotypic variations in pathology observed within the same class of neurodegenerative maladies, if one ignores conformational diversity of aggregated states and the possibility that each of these states exhibit the capacity of recruiting a variety of cytotoxic mechanisms. Nevertheless, extrapolating the relationships between molecular structure, size and cytotoxicity observed in cultured cells to the pathology *in vivo* needs to be considered with great caution because glial cells and astrocytes might neutralize aggregates of a certain size, protecting neuronal cells, or become activated and inflamed leading to additional neuronal damage [39]–[41]. Considering that silencing of PrP^C^ expression by shRNA did not restore cell viability completely (Fig. 4B), extracellular PrP fibrils might also trigger PrP^C^-independent cytotoxic effects. This result is consistent with previous findings where extracellular PrP fibrils or oligomers were shown to exhibit toxicity in a PrP^C^-independent manner in primary neurons or animals [7]. Because PrP^C^ silencing abolished the toxic effect of R- and S-structures to a different extent, the role of PrP^C^-mediated versus PrP^C^ -independent signaling pathways appears to depend on the structure of toxic aggregates.

While the relationship between fibrillar size and their pathogenic activity were opposite for R- and S-structures, both amyloid structures were found to reduce mitochondrial activity significantly (Fig. 3B). In the last several years, a growing body of evidence has emerged suggesting that protein deposits, including aggregates of Aβ and PrP, cause mitochondrial dysfunctions including inhibition or modification of the mitochondrial respiratory complex and deleterious alterations in mitochondrial morphology [33], [42]–[44]. During progression of prion diseases, functional abnormalities in mitochondria were observed in brain areas with substantial synaptic pathology, which is considered to be a key early sign in prion diseases, suggesting that a link exists between the two abnormalities [42], [45]. Previous studies revealed that treatment of primary neuronal cultures with rPrP or Aβ fibrils caused axonal degeneration and formation of beads composed of aggregated cytoskeletal and motor proteins as a result of an impairment of the neuronal transport system [39], [43]. Furthermore, in rodent models of the prion diseases, severe axonal transport defects were found to accompany the progression of the diseases [46], [47].

In summary, this work revealed a deficiency in the current concept about the relationship between the physical dimension and cytotoxic potential of ordered protein aggregates. This study demonstrates that the molecular structure controls the direction in which the cytotoxic potential of ordered protein aggregates changes with the change in their physical dimension. This work helps to find a common ground for conflicting data on size and toxicity of protein aggregates.

## Materials and Methods

### Preparation of rPrP fibrils

Syrian hamster full-length rPrP (residues 23–231) was expressed and purified as previously described [48]. The fibrillation reactions were conducted in 2 M GdnHCl, 50 mM MES, pH 6.0 at 37°C at slow agitation (∼60 rpm) and rPrP concentration of 0.25 mg/ml for producing R-fibrils or at rapid shaking (∼1000 rpm) and rPrP concentration of 0.5 mg/ml for producing S-fibrils. The yield of conversion to fibrillar forms was estimated by SDS-PAGE as previously described [49] and was found to be >99%. For each preparation, the R- or S-specific fibrillar features were confirmed by AFM and FTIR as described [18], [19]. To prepare fibrillar fragments, fibrils were subjected to ultrasound treatment for 1 min in a bath sonicator (Bransonic-2510, Danbury, CT) as previously described [12]. R- or S-fibrillar fragments were structurally different from the previously described soluble β-oligomeric rPrP particles, which are formed at acidic pH through an aggregation pathway different from fibrillation [14].

### Transfection of CHO cells and analysis of PrP^C^ expression

FLP-In™ CHO cells (Invitrogen, Carlsbad, CA) cells were transfected with pcDNA5/FRT/PrP plasmid (Invitrogen, Carlsbad, CA) containing the gene for expression of full-length wild type Syrian hamster PrP^C^. pcDNA5/FRT/PrP plasmid was constructed and used for transfection as previously described [50]. Consistent with previous studies [25], [50], expression of endogenous PrP^C^ in non-transfected CHO cells was found to be below detectible levels, whereas the CHO cells transfected with pcDNA5/FRT/PrP plasmid expressed PrP^C^ in amounts comparable to those found in normal hamster brains (Fig. 2). FLP-In™ CHO cells before and after transfection and SKMEL-2 (American Type Culture Collection, Manassas, VA) and SKMEL-28 cells (American Type Culture Collection ) (10^7^ cells for each cell line) were lysed in ProteoJET Mammalian Cell Lysis Reagent (Fermantas, Glen Burnie, MD) with a protease inhibitor cocktail (Roche Diagnostics, Indianapolis, IN). After centrifugation at 16,000×g for 15 min at 4°C, the supernatant was transferred to a new tube and protein concentrations were determined by absorbance spectroscopy. Twelve micrograms of total protein were loaded to 12% SDS-PAGE, and then analyzed by Western blotting using mouse anti-PrP 3F4 or mouse anti-β-actin antibodies (Sigma-Aldrich, Saint Louis, MO).

### Cytotoxicity Assays

The cytotoxic potential of rPrP fibrils was assessed using Chinese hamster ovary cell (CHO) or human melanoma SKMEL cell lines. CHO or SKMEL lines were cultured in F-12 medium (Invitrogen, Carlsbad, CA) or RPMI 1640 (Invitrogen), respectively, both supplemented with 10% fetal bovine serum (Invitrogen) in a humidified atmosphere of 5% CO_2_, 95% air at 37°C. Intact or fragmented fibrils were added to the cultured cells at concentrations equivalent to 1 µM (as calculated per concentration of rPrP molecules) and incubated for 24 h. The cytotoxic effect was analyzed using Trypan Blue (Mediatech, Manassas, VA) or XTT (Sigma-Aldrich, Saint Louis, MO) assays according to the manufacturer's procedure. Briefly, for the Tryptan Blue assay, after treatment with fibrils, cells were trypsinized, incubated with 0.2% trypan blue solution (Mediatech, Manassas, VA) for 2 minutes, and counted using light microscopy. For the XTT assay, XTT (2,3-Bis (2-methoxy-4-nitro-5-sulfophenyl)-2H-tetrazolium-5-carboxanilide) was added to the amount equal to 20% of the culture medium volume, the cells were then incubated for four hours in 5% CO_2_ at 37°C, and the dehydrogenase activity was measured by monitoring absorbance at 450 and 690 nm according to the manufacturer's procedure.

### Construction of lentiviral-derived vectors carrying shRNA-PrP^C^

Target sequences were derived from the 3′ UTR region of human *Prnp* gene (GenBank BC022532). CAATAGGGAGACAATCTAA (1899-1917, sequence #1) and GCAATGTTATTATTGGCTT (2054–2072, sequence #2) were selected as target sequences for silencing the expression of PrP^C^, the scrambled sequence GAATGCAATAACGAGAGTA was used as a negative control for testing the effects of non-specific shRNA. To avoid off-target effects, a homology search was performed using BLAST (http://blast.ncbi.nlm.nih.gov) to ensure that only the PrP^C^ mRNA sequence was targeted. Two complementary oligonucleotides necessary to create the hairpin insert for pENTR-H1/TO vectors were designed using SiRNA Scales software [51]. The following single stranded oligonucleotides were synthesized:

shRNA-PrP^C^#1-Top, 5′-CACCGAATAGGGAGACAATCTAACGAATTAGATTGTCTCCCTATTC-3′

shRNA-PrP^C^#1-Bot., 5′-AAAAGAATAGGGAGACAATCTAATTCGTTAGATTGTCTCCCTATTC-3′

shRNA-PrP^C^#2-Top, 5′-CACCGCAATGTTATTATTGGCTTCGAAAAGCCAATAATAACATTGC-3′ shRNA-PrP^C^#2-Bot., 5′-AAAAGCAATGTTATTATTGGCTTTTCGAAGCCAATAATAACATTGC-3′ shRNA-Scrmbl-Top, 5′-CACCGAATGCAATAACGAGAGTACGAATACTCTCGTTATTGCATTC-3′ shRNA-Scrmbl-Bot., 5′-AAAAGAATGCAATAACGAGAGTATTCGTACTCTCGTTATTGCATTC-3′

After annealing, each double-stranded oligonucleotide (5 nM) was cloned into pENTR/H1/TO vector (Invitrogen, Carlsbad, CA). To construct shRNA-expression vectors, recombination reactions of pENTR/H1/TO vectors that included specific target sequences with pLenti4/BLOCK-iT-DEST vector (Invitrogen, Carlsbad, CA) were performed. For producing lentivirus, the resulting pLenti4/BLOCK-iT-DEST vectors were mixed with ViraPower Packaging Mix (Invitrogen, Calrsbad, CA), transfected into 293FT cells (6×10^6^), and virus-containing supernatants were harvested 72 hours post-transfection. The lentiviruses were added to the SKMEL-28 cell line at a multiplicity of infection (MOI) of 2–5, and cells were cultured using a complete medium containing 50 µg/ml Zeocin (Invitrogen, Carlsbad, CA) for 14 days to establish stable cell lines.

## References

[1] Carrell RW, Lomas DA (1997). Conformational disease.. Lancet.

[2] Prusiner SB (1997). Prion diseases and the BSE crisis.. Science.

[3] Caughey B, Lansbury PT (2003). Protofibrils, pores, fibrils, and neurodegeneration: separating the responsible protein aggregates from the innocent bystanders.. Annu Rev Neurosci.

[4] Aguzzi A, Heikenwalder M, Polymenidou M (2007). Insights into prion strains and neurotoxiciy.. Nat Rev Mol Cell Biol.

[5] Kirkitadze MD, Bitan G, Teplow DB (2002). Paradigm shifts in Alzheimer's disease and other neurodegenerative disorders: the emerging role of oligomeric assemblies.. J Neurosci Res.

[6] Xue W-F, Hellewell AL, Hewitt EW, Radford SE (2010). Fibril fragmentation in amyloid assembly and cytotoxicity.. Prion.

[7] Simoneau S, Rezaei H, Sales N, Kaiser-Schultz G, Lefebvre-Roque M (2007). In Vitro and In Vivo Neurotoxicity of Prion Protein Oligomers.. PLOS Pathog.

[8] Kirkitadze MD, Condron MM, Teplow DB (2001). Identification and characterization of key kinetic intermediates in amyloid beta-protein fibrillogenesis.. J Mol Biol.

[9] Kaylor J, Bodner N, Edridge S, Yamin G, Hong DP (2005). Characterization of Oligomeric Intermediates in alpha-Synuclein Fibrillation: FRET Studies of Y125W/Y133F/Y136F alpha-Synuclein.. J Mol Biol.

[10] Zhu M, Han S, Zhou F, Carter SA, Fink AL (2004). Annular olgomeric amyloid intermediates observed by in situ atomic force microscopy.. J Biol Chem.

[11] Bitan G, Kirkitadze MD, Lomakin A, Vollers SS, Benedek GB (2002). Amyloid beta-protein (Abeta) assembly: Abeta 40 and Abeta 42 oligomerize through distinct pathways.. Proc Acad Natl Sci U S A.

[12] Sun Y, Makarava N, Lee CI, Laksanalamai P, Robb FT (2008). Conformational stability of PrP amyloid firbils controls their smallest possible fragment size.. J Mol Biol.

[13] Baskakov IV, Legname G, Baldwin MA, Prusiner SB, Cohen FE (2002). Pathway Complexity of Prion Protein Assembly into Amyloid.. J Biol Chem.

[14] Bocharova OV, Breydo L, Parfenov AS, Salnikov VV, Baskakov IV (2005). In vitro conversion of full length mammalian prion protein produces amyloid form with physical property of PrPSc.. J Mol Biol.

[15] Souillac PO, Uversky VN, Millett IS, Khurana R, Doniach S (2005). Elucidation of the molecular mechanism during the early events in immunoglobulin light chain amyloid fibrillation. Evidance for an off-pathway oligomer at acidic pH.. J Biol Chem.

[16] Silveira JR, Raymond GJ, Hughson A, Race RE, Sim VL (2005). The most infectious prion protein particles.. Nature.

[17] Bucciantini M, Giannoni E, Chiti F, Baroni F, Formigli L (2002). Inherent toxicity of aggregates implies a common mechanism for protein misfolding diseases.. Nature.

[18] Makarava N, Baskakov IV (2008). The same primary structure of the prion protein yields two distinct self-propagating states.. J Biol Chem.

[19] Ostapchenko VG, Sawaya MR, Makarava N, Savtchenko R, Nilsson KP (2010). Two amyloid states of the prion protein display significantly different folding patterns.. J Mol Biol.

[20] Novitskaya V, Bocharova OV, Bronstein I, Baskakov IV (2006). Amyloid Fibrils of Mammalian Prion Protein Are Highly Toxic to Cultured Cells and Primary Neurons.. J Biol Chem.

[21] Brandner S, Isenmann S, Raeber A, Fischer M, Sailer A (1996). Normal host prion protein necessary for scrapie-induced neurotoxicity.. Nature.

[22] Mallucci G, Dickinson A, Linehan J, Klohn PC, Brandner S (2003). Depleting Neuronal PrP in Prion Infection Prevents Disease and Reverses Spongiosis.. Science.

[23] Radford HE, Mallucci GR (2009). The role of GPI-anchored PrP C in mediating the neurotoxic effect of scrapie prions in neurons.. Curr Issues Mol Biol.

[24] Mallucci GR, White MD, Farmer M, Dickinson A, Khatun H (2011). Targeting cellular prion protein reverses early cognitive deficits and neurophysiological dysfunction in prion-infected mice.. Neuron.

[25] Blochberger TC, Cooper C, Peretz D, Tatzelt J, Griffith OH (1997). Prion protein expression in Chinese hamster ovary cells using a glutamine synthetase selection and amplification system.. Prot Eng.

[26] Tycko R, Savtchenko R, Ostapchenko VG, Makarava N, Baskakov IV (2010). The a-Helical C-Terminal Domain of Full-Length Recombinant PrP Converts to an In-Register Parallel β-Sheet Structure in PrP Fibrils: Evidence from Solid State Nuclear Magnetic Resonance.. Biochemistry.

[27] Makarava N, Kovacs GG, Bocharova OV, Savtchenko R, Alexeeva I (2010). Recombinant prion protein induces a new transmissible prion disease in wild type animals.. Acta Neuropathol.

[28] Bosque PJ, Ryou C, Telling G, Peretz D, Legname G (2002). Prions in skeletal muscle.. Proc Acad Natl Sci U S A.

[29] Mabbott N, Turner M (2005). Prions and the blood and immune systems.. Haematologica.

[30] Aguzzi A (2003). Prions and the immune system: a journey through gut, spleen, and nerves.. Adv Immunol.

[31] Herzog C, Salès N, Etchegaray N, Charbonnier A, Freire S (2004). Tissue distribution of bovine spongiform encephalopathy agent in primates after intravenous or oral infection.. Lancet.

[32] Krasemann S, Neumann M, Geissen M, Bodemer W, Kaup FJ (2010). Preclinical deposition of pathological prion protein in muscle of experimentally infected primates.. Plos ONE.

[33] Resenberger UK, Harmeier A, Woerner AC, Goodman JL, Müller V (2011). The cellular prion protein mediates neurotoxic signalling of ß-sheet-rich conformers independent of prion replication.. EMBO J.

[34] Lauren J, Gimbel DA, Nygaard HB, Gilbert JW, Strittmatter SM (2009). Cellular prion protein mediates impairment of synaptic plasticity by amyloid-beta oligomers.. Nature.

[35] Gimbel DA, Nygaard HB, Coffey EE, Gunther EC, Lauren J (2010). Memory impairment in transgenic Alzheimer mice requires cellular prion protein.. J Neurosci.

[36] Harris DA, True HL (2006). New insights into prion structure and toxicity.. Neuron.

[37] Parchi P, Giese A, Capellari S, Brown P, Schulz-Schaeffer W (1999). Classification of sporadic Creutzfeldt-Jakob disease based on molecular and phenotypic analysis of 300 subjects.. Ann Neurol.

[38] Hill AF, Joiner S, Wadsworth JDF, Sidle KCL, Bell JE (2003). Molecular classification of sporadic Creutzfeldt-Jakob disease.. Brain.

[39] Novitskaya V, Makarava N, Sylvester I, Bronstein IB, Baskakov IV (2007). Amyloid fibrils of mammalian prion protein induce axonal degeneration in NTERA2-derived terminally differentiated neurons.. J Neurochem.

[40] Brown GC (2007). Mechanisms of inflammatory neurodegeneration: iNOS and NADPH oxidase.. Biochem Soc Trans.

[41] Thellung S, Corsaro A, Villa V, Venezia V, Nizzari M (2007). Amino-terminally truncated prion protein PrP90-231 induces microglial activation in vitro.. Ann N Y Acad Sci.

[42] Siskova Z, Mahad DJ, Pudney C, Asuni A, O'Connor V (2010). Morphological and Functional Abnormalities in Mitochondria Associated with Synaptic Degeneration in Prion Disease.. Am J Pathol.

[43] Takeuchi H, Mizuno T, Zhang G, Wang J, Kawanokuchi J (2005). Neuritic beading induced by activated microglia is an early feature of neuronal dysfunction toward neuronal death by inhibition of mitochondrial respiration and axonal transport.. J Biol Chem.

[44] Park JH, Kim BH, Park SJ, Jin JK, Jeon YC (2010). Association of endothelial nitric oxide synthase and mitochondrial dysfunction in the hippocampus of scrapie-infected mice.. Hippocampus.

[45] Cunningham C, Deacon R, Wells H, Boche D, Waters S (2003). Synaptic changes characterize early behaavioral signs in the ME7 model of murine prion disease.. Eur J Neurosci.

[46] Ermolayev V, Cathomen T, Merk J, Friedrich M, Hartig W (2009). Impaired Axonal Transport in Motor Neurons Correlates with Clinical Prion Disease.. PLOS Pathog.

[47] Ermolayev V, Fredrich M, Nozardze R, Cathomen T, Klein MA (2009). Ultramicroscopy Reveals Axonal Transport Impairments in Cortical Motor Neurons at Prion Disease.. Biophys J.

[48] Ostapchenko VG, Makarava N, Savtchenko R, Baskakov IV (2008). The polybasic N-terminal region of the prion protein controls the physical properties of both the cellular and fibrillar forms of PrP.. J Mol Biol.

[49] Makarava N, Lee CI, Ostapchenko VG, Baskakov IV (2007). Highly promiscuous nature of prion polymerization.. J Biol Chem.

[50] Geoghegan JC, Miller MB, Kwak AH, Harris BT, Supattapone S (2009). Trans-Dominant Inhibition of Prion Propagation In Vitro Is Not Mediated by an Accessory Cofactor.. PLOS Pathog.

[51] Matveeva O, Nechipurenko Y, Rossi L, Moore B, Saetrom P (2007). Comparison of approaches for rational siRNA design leading to a new efficient and transparent method.. Nucl Acids Res.

